# The Association Between Atypical Sensory Processing in Adults with Mental Illness and Occupational Participation and Engagement: A Scoping Review

**DOI:** 10.3390/brainsci16101037

**Published:** 2026-09-28

**Authors:** Tawanda Machingura, Carly Hudson, Lauren Waters

**Affiliations:** Faculty of Health Sciences and Medicine, Bond University, Gold Coast, QLD 4226, Australia; tmaching@bond.edu.au (T.M.); chudson@bond.edu.au (C.H.)

**Keywords:** sensory processing, sensory profile, sensory modulation, mental illness, schizophrenia, participation, occupational engagement, functional performance, quality of life, occupational therapy

## Abstract

**Highlights:**

**What are the main findings?**

- Atypical sensory processing represents a potentially modifiable contributor to participation-related difficulties experienced by people with a mental illness.
- Low registration, sensory sensitivity, and sensory avoidance were associated with lower occupational engagement, social participation difficulties, and reduced quality of life.
- Associations between atypical sensory processing and participation-related outcomes are seen when sensory processing is measured subjectively and objectively.

**What are the implications of the main findings?**

- Findings suggest that health professionals supporting adults with mental illness should assess sensory processing; they should ask not only “What symptoms prevent participation?” but also “What sensory experiences make participation difficult?” as atypical sensory processing may be modulated to support adults experiencing mental illness to participate and engage in meaningful occupations.
- Greater conceptual and terminological consistency should be agreed upon to facilitate trans-disciplinary communication.

**Abstract:**

**Background/Objectives**: Atypical sensory processing in adults experiencing mental illness has been widely studied. The purpose of this review is to systematically map and synthesise literature examining the relationship between atypical sensory processing and participation-related outcomes in adults experiencing mental illness. **Methods**: A scoping review was conducted in accordance with the Joanna Briggs Institute methodology and the PRISMA-ScR reporting guidelines. Six electronic databases (MEDLINE via PubMed, Embase, Web of Science, CINAHL, Scopus, and the Cochrane Library) were searched from inception to March 2026. Additional grey literature sources were searched via ProQuest. Eligible studies recruited adults experiencing mental illness, reported on sensory processing, and included outcomes relating broadly to participation. **Results**: Eighteen studies met the inclusion criteria; most focused on schizophrenia spectrum disorders (*n* = 10). Across diagnoses, participants demonstrated higher rates of atypical sensory processing than normative or control populations. Studies using the Adult/Adolescent Sensory Profile indicated that low registration, sensory sensitivity, and sensory avoidance were associated with reduced participation-related outcomes. Studies using objective measures to identify atypical sensory processing similarly found associations with poorer functioning and quality of life, although relationships were often mediated by other variables. Qualitative studies described the influence of sensory experiences on participation and the use of self-management strategies to support occupational engagement. Evidence for sensory modulation interventions was limited but suggested potential improvements in participation. **Conclusions**: Atypical sensory processing may negatively impact participation in adults experiencing mental illness. Sensory processing represents a potentially modifiable contributor to participation-related outcomes. Further research is required to clarify causal relationships, establish intervention effectiveness, and develop greater conceptual and terminological consistency across disciplines.

## 1. Introduction

The International Classification of Functioning Disability and Health (ICF) defines participation as involvement in a life situation [1]. Participation in everyday activities facilitates the development of skills and competencies that support functional performance, while meaningful participation that is sufficiently challenging, goal-directed, and grounded in choice and control promotes health and wellbeing [2]. Participation occurs within an environment; the dynamic interpretation of contextual factors through sensory functions can support or hinder participation [1,3]. Sensory processing is the mechanism through which individuals interpret and respond to the environment and their own internal bodily structures. Sensory input is processed (coded, interpreted, and modulated) alongside other cognitive functions (e.g., attention, memory) and in response to task demands [4,5]. Aligning an individual’s sensory processing needs with environmental and occupational demands supports occupational engagement [6]. Atypical sensory processing may influence how individuals experience occupations, their level of engagement in everyday activities, and the ways they participate across home, community, work, and social contexts [6].

Sensory processing patterns vary between individuals and can differ significantly in people with various disabilities or health conditions [5]. This variation is shaped by two factors: an individual’s neurological threshold for sensory input and the active or passive self-regulation strategies they use to respond to and manage sensory experiences. When sensory thresholds are either unusually high or low, or when regulatory strategies are ineffective or poorly matched to environmental or task demands, sensory processing becomes atypical, and individuals may experience difficulties that adversely affect participation [5,7]. Atypical sensory processing has been reported at elevated rates across a range of mental illnesses, and given the importance of participation to health, wellbeing, and recovery, this review focuses on the relationship between atypical sensory processing and participation in adults experiencing mental illness [8].

Atypical sensory processing in people experiencing mental illness has been reported in research within the fields of neuroscience, psychology, and occupational therapy using both objective and subjective measures [8]. Objective studies of neurobiological processes have found that people experiencing mental illness demonstrate atypical neural responses in sensory gating (i.e., filtering out irrelevant information in a sensory-rich environment); deficits in auditory and visual processing are also clearly established [8,9]. In the occupational therapy literature, people experiencing mental illness have repeatedly been found to report atypical sensory experiences on standardized self-report measures with known psychometric properties such as the Adult/Adolescent Sensory Profile (A/ASP; [10]), Sensory Processing Measure [11], and Sensory Responsiveness Questionnaire [12]. Atypical sensory processing, whether measured objectively or subjectively, has been associated with reduced participation, either directly or through a mediating variable such as social cognition [9,13,14]. Participation in meaningful occupations is a key component of recovery-oriented mental health practice, which prioritizes autonomy, community inclusion, self-determination, and engagement in valued life roles [15]. Given that sensory processing may shape how individuals access and engage in everyday occupations, a better understanding of its relationship with participation may identify additional avenues for supporting recovery alongside traditional symptom-focused interventions.

Research that explicitly explores the relationship between sensory processing and participation is limited, and synthesis of the extant literature is challenging due to inconsistencies in terminology across disciplines [14]. However, the importance of sensory processing to participation and recovery suggests that occupational therapy should expand research on this phenomenon to develop more tailored interventions to support people experiencing mental illness [8,16]. Previous reviews have primarily focused on the relationship between sensory processing and participation in people with schizophrenia spectrum disorders [9,13,14]. Only one scoping review has directly examined the relationship between atypical sensory processing and participation, finding mixed results across five studies and identifying ambiguity and inconsistencies in terminology as a barrier to synthesis and interdisciplinary research [14]. Related reviews have examined adjacent constructs, including the mediating role of social cognition between early visual processing and functional outcomes [9], and the effectiveness of sensory modulation interventions for reducing distress [13]. Together, this literature is limited in volume and confined to schizophrenia spectrum disorders, leaving the relationship between atypical sensory processing and participation in the broader mental illness population unexamined.

This scoping review, therefore, aims to systematically map what is known about the relationship between atypical sensory processing and participation in adults experiencing mental illness through identifying and synthesizing data from peer-reviewed quantitative studies (observational or analytical) and qualitative studies. Participation-related outcomes were conceptualized broadly to encompass not only participation as defined in the ICF [1], but also related outcomes such as occupational engagement, occupational and functional performance, and quality of life. Within occupational therapy and rehabilitation, these constructs are closely interconnected, with performance supporting participation, engagement reflecting involvement in occupation, and quality of life representing a broader indicator of participation experiences and outcomes. Given the inconsistent use of participation-related terminology within the sensory processing and mental health literature [14], a broad conceptualization was adopted to capture the full scope of relevant evidence.

## 2. Methods

This scoping review followed the JBI methodology for scoping reviews [17] and is reported in accordance with the PRISMA-ScR reporting guidelines [18]. The review was conducted in accordance with an a priori protocol registered on the Open Science Framework. This review was guided by the following PCC framework (population: adults diagnosed with serious mental illness; Concept/s: sensory processing and participation; Context: all healthcare and community settings).

### 2.1. Eligibility Criteria

Peer-reviewed papers published in English from inception to the search date (12 March 2026) were eligible for inclusion if participants were aged over 18 years (adult), the population or treatment group had been diagnosed with an Axis I mental health condition, sensory function (i.e., sensory processing, sensory modulation, sensory gating) was a key concept addressed in the study, and outcomes included participation, occupational engagement, functional performance, or quality of life. Quantitative and qualitative study designs were eligible for inclusion. Case studies, case series, reviews, and conference abstracts were excluded because they were deemed less likely to provide the information required to answer the research question. Two relevant theses were identified through grey-literature searching.

### 2.2. Search Strategy

The initial search strategy was developed with the assistance of a health librarian and carried out in MEDLINE via PubMed. Subsequently, search terms were refined using the Polyglot Search Translator, SearchRefiner, MeshMate, and Word Freq tools in The Evidence Review Accelerator (TERA) suite [19,20]. A second search was then conducted across Embase (Elsevier), Web of Science, CINAHL (Ebsco), Scopus (basic search), Cochrane Library and ProQuest on 12 March 2026 using MeSH or other subject terms, synonyms, and search filters (English). Search terms included (“sensory modulation” OR “sensory processing” OR “processing patterns” OR “sensory profile” OR “low registration” OR “sensory sensitivity”) AND (“mental health” OR “mental disorders” OR “mental health” OR “mental illness”) AND (“activities of daily living” OR “occupational engagement” OR “occupational participation” OR “occupational therap*” OR “daily life” OR occupation*” OR “quality of life”). Authors (LW, TM, CH) removed duplicates from the results using the Deduplicator tool in the TERA suite [21].

### 2.3. Screening and Data Extraction

Review authors (TM, LW, CH) independently screened the titles and abstracts against the inclusion criteria using the Screenatron tool in the TERA suite [19]. Following initial title and abstract screening, backward and forward citation analysis of included articles was performed using the SpiderCite tool. The citation search was screened by LW and CH. For articles eligible after screening, full texts were retrieved by LW and reviewed by LW and CH using Covidence. Discrepancies were resolved by referring to a third author (TM). The screening process is summarized in Figure 1.

Data extraction was conducted by LW using a data extraction form for study characteristics and outcomes, which was piloted across four studies and reviewed by TM. The following data for study characteristics and outcomes were extracted from each included study:

- Author(s), year of publication, country of origin;
- Aim/research question;
- Population and sample size;
- Methodology;
- Measurement and outcomes;
- Key findings in relation to the scoping review aim.

## 3. Results

Database searching identified 1270 references which were imported into TERA; 672 duplicate references were identified and removed. An additional 52 references were located through forward and backward citation searching. Titles and abstracts from 650 references were screened, with 105 progressing to full-text screening in Covidence. Of these, 18 met the inclusion criteria and were included in the review (see Figure 1).

### 3.1. Characteristics of Included Studies

The literature search did not identify any relevant articles published prior to 2005; the included studies were published between 2005 and 2025. Of the 18 primary research studies, two were qualitative, two were quasi-experimental, and the remaining 14 used a cross-sectional design. Over half of the included studies (10/18) specifically addressed participants diagnosed with schizophrenia or schizophrenia spectrum disorders. Other populations included post-traumatic stress disorder (*n* = 1), mixed serious mental illness (*n* = 5), affective disorders including depression and bipolar disorder (*n* = 1), and anxiety disorders (*n* = 1).

Studies were conducted across North America, Europe, the Middle East, Asia, Australia and New Zealand. Sample sizes ranged from 4 to 267 participants. The characteristics of included studies are presented in Table 1.

### 3.2. Sensory Processing Is Atypical in People Experiencing Mental Illness: Comparisons with a Normative Population Group or Control

Studies comparing adults with mental illness to normative or control populations found differences in subjective measures of sensory processing across diagnostic groups. In a mixed psychiatric sample, Forsberg et al. [26] found a significantly higher proportion of participants reporting “much more than most people” on sensory sensitivity (38–53%), sensory avoidance (13–51%), and low registration (13–41%) on the A/ASP compared to a normative population; Forsberg et al. [26] additionally reported significantly lower proportion of participants reporting “much more than most people” for sensation seeking (0%) relative to normative values. Similarly, Machingura et al. [32] found significantly higher low registration (*p* = 0.01) and sensation-avoiding (*p* = 0.01) scores among people with schizophrenia spectrum disorders compared with normative A/ASP data. Halperin et al. [28], also studying a sample with schizophrenia spectrum disorders, reported that a majority of participants scored outside the normative range on all four A/ASP quadrants (low registration, 70.6%; sensation seeking, 64.7%; sensory sensitivity, 58.8%; sensory avoidance, 58.8%) but did not specify the direction of difference for each quadrant, limiting comparison with the directional findings reported elsewhere. Lipskaya-Velikovsky et al. [31] found significant differences between patients with schizophrenia and controls on the Sensory Responsiveness Questionnaire (SRQ; *p* < 0.001), indicating under-responsivity and a trend toward dysfunction in participants with schizophrenia. Significant differences between participants with schizophrenia and controls were also reported on objective measures of sensory processing. Brittain et al. [24] found that patients performed worse than controls on 2 out of 4 visual processing tasks (masking *p* = 0.048; biological motion *p* = 0.02), while Light & Braff [30] found that participants had significantly smaller mismatch response amplitudes than controls, but comparable topographic distributions when amplitude differences were corrected. Together, these findings suggest that atypical sensory processing is not restricted to a single diagnostic category but appears across multiple psychiatric populations; however, the way in which “difference” was operationalized was not consistent across studies, and this variability is a notable feature of the current evidence base.

### 3.3. Sensory Processing Is Associated with Participation

#### 3.3.1. Cross-Sectional Studies Reporting on Participation

Across studies examining participation and occupational engagement, atypical sensory processing was generally associated with reduced participation, although the strength and direction of these relationships varied by sensory processing pattern and clinical population. A consistent finding across diagnostic groups was that low registration and sensory sensitivity were associated with poorer participation outcomes. Forsberg et al. [26] found that higher levels of low registration and sensory sensitivity, respectively, were correlated with lower occupational engagement in a mixed population of mental health service users (r_s_ = −0.263, *p* ≤ 0.01; r_s_= −0.328, *p* ≤ 0.01). People with serious mental illness in the “more or much more than most” category for low registration and sensory sensitivity reported statistically significant reductions in participation variables compared to those in the “similar to, less than, or much less than most” category (*p* = 0.0007–*p* = 0.0168) [16]. Greater social participation difficulties in a population with serious mental illness were correlated with low registration (r_s_ = 0.53, *p* ≤ 0.01) and sensory sensitivity (r_s_ = 0.63, *p* ≤ 0.01) [29].

Sensory avoidance also emerged as a consistent correlate of participation restrictions, being associated with lower occupational engagement (r_s_ = −0.425, *p* ≤ 0.001) [26] and greater social participation difficulties (r_s_ = 0.82, *p* ≤ 0.01) [29]. People with PTSD and atypical sensory avoidance also reported reduced participation intensity (*p* = 0.015), diversity (*p* = 0.018), and meaning (*p* = 0.000) compared to those with typical sensory avoidance [37]. Notably, Hattori et al. [29] identified sensory avoidance as a potential explanatory mechanism, demonstrating that it fully mediated the relationship between respiratory sinus arrhythmia and social participation.

Conversely, higher levels of sensation seeking were associated with greater occupational engagement (r_s_ = 0.362, *p* ≤ 0.001; [26]) and people in the “similar to, more than, or much more than most people category” category for sensory seeking reported significantly higher scores in participation variables compared to those not in those categories (*p* = 0.0027–*p* = 0.0036; [16]). While this may suggest that sensation seeking is associated with greater opportunities for participation, this relationship was not found consistently across studies, limiting conclusions regarding its broader significance.

Despite the overall pattern linking atypical sensory processing with reduced participation, some findings suggest that sensory processing may not be the primary determinant of participation outcomes. For example, although Lipskaya-Velikovsky et al. [31] reported greater sensory under-responsivity and lower participation among people with schizophrenia compared with healthy controls, sensory responsiveness did not independently predict participation; cognitive functioning and negative symptom severity were significant predictors of participation, explaining 14 to 40% of variance. Similarly, Shapira et al. [37] found that only sensory avoidance, and not low registration or sensory sensitivity, was associated with participation outcomes in PTSD. Together, these findings suggest that while sensory processing is an important correlate of participation, its influence may be moderated by condition-specific factors and may operate alongside other determinants, such as cognitive functioning, symptom severity, and physiological regulation.

#### 3.3.2. Cross-Sectional Studies Reporting on Functional Performance

Across studies examining functional performance and functioning, sensory processing deficits were generally associated with poorer functional outcomes, particularly among people with schizophrenia. However, the relationship was rarely direct. A particularly consistent finding was the mediating role of social cognition and related higher-order processes. Brittain et al. [24] found that visual-processing deficits exerted only a small indirect effect on role functioning through social cognition, accounting for only 3% of the variance in functioning. Similarly, Sergi et al. [36] demonstrated that the relationship between early visual processing and functional status was fully mediated by social perception, while Green et al. [27] extended this model by identifying a pathway from visual processing to functioning via social cognition and beliefs/motivation. The importance of indirect mechanisms was further reinforced by Rassovsky et al. [34], who found that negative symptoms were the strongest predictor of functional status and fully mediated the relationship between visual processing and functioning. Collectively, these findings indicate that associations between sensory processing and functioning are often mediated by social, cognitive, motivational, and clinical factors.

Evidence from studies examining other aspects of sensory processing also supported possible associations with objectively measured functional performance. Halperin and Falk-Kessler [28] reported a moderate positive correlation between sensory avoidance and increased motor skill deficits (r = 0.591, *p* = 0.072) during activities of daily living, though this was not statistically significant. Similarly, Light and Braff [30] found that greater mismatch negativity impairment was associated with poorer global functioning, accounting for up to 42% of variance in functional status. Notably, mismatch negativity was not associated with functional capacity as measured by the UPSA, indicating that sensory-processing deficits may be more closely related to real-world functional performance than to performance under structured assessment conditions.

#### 3.3.3. Cross-Sectional Studies Reporting on Quality of Life

Across studies examining quality of life (QoL), atypical sensory processing was generally associated with poorer QoL outcomes, although the strength and nature of these relationships varied according to sensory processing pattern, QoL domain, and diagnostic group. The most consistent findings were observed for low registration, sensory sensitivity, and sensory avoidance, which were associated with reduced QoL across multiple studies. In people with schizophrenia, Sanchis-Assissi et al. [35] found significant negative correlations between quality of life and low registration (r = −0.225, *p* < 0.01), sensory sensitivity (r = −0.206, *p* < 0.01), and sensory avoidance (r = −0.219, *p* < 0.01), with regression analyses demonstrating distinct associations between sensory processing variables and QoL domains. Greater sensory sensitivity and auditory processing difficulties were associated with poorer physical health, while low registration and auditory processing difficulties were associated with poorer psychological health. In contrast, sensory seeking was positively associated with psychological health and social relationships. Similarly, Engel-Yeger et al. [25] reported multiple significant correlations between low registration, sensory sensitivity, sensory avoidance, and reduced scores on QoL domains in people with mood disorders, although these relationships were more frequently observed among individuals with unipolar than bipolar disorders. Notably, although coping remained the strongest predictor of mental QoL, sensory sensitivity accounted for 6% of the variance, while low registration accounted for 4% of the variance in physical quality of life.

Evidence from studies examining objective sensory-processing deficits further supported an association with reduced QoL. Micoulaud-Franchi et al. [33] found that adults with schizophrenia who demonstrated sensory gating deficits reported greater perceptual difficulties and poorer psychological wellbeing than those without sensory gating deficits. These associations remained significant after adjustment for multiple potential confounders. Collectively, the evidence suggests that both subjective sensory-processing patterns and objective sensory-processing deficits are associated with poorer QoL, particularly in the psychological and social domains.

### 3.4. The Impact of Sensory Modulation Interventions on Participation-Related Variables: Quasi-Experimental Studies

Two quasi-experimental studies examined the impact of sensory modulation interventions on participation-related outcomes in clinical populations. Both studies reported improvements in occupational performance and participation-related outcomes following intervention, although the strength of evidence supporting a treatment effect was limited. In people with schizophrenia, Machingura et al. [32] reported significant within-group improvements in occupational performance, distress, and health and social functioning, with moderate-to-large effect sizes. However, the absence of significant between-group differences or group-by-time interactions indicated that these improvements could not be attributed specifically to the intervention. Similarly, Wallis et al. [38] reported improvements in occupational performance, occupational satisfaction, disability, and state anxiety among individuals with anxiety disorders participating in a sensory modulation programme. Qualitative findings further suggested that participants experienced greater control over sensory experiences and increased confidence in managing daily activities. However, the small sample size (*n* = 4) and variable quantitative outcomes limited the strength of conclusions that could be drawn regarding effectiveness. Collectively, the available quasi-experimental evidence suggests that sensory modulation interventions may be associated with improvements in participation-related outcomes, although evidence for a causal effect remains limited.

### 3.5. The Influence of Sensory Experiences on Everyday Participation: Qualitative Studies

Across qualitative studies, sensory experiences were described as a significant influence on everyday participation, shaping both opportunities for and barriers to occupational engagement. Participants consistently reported that sensory challenges could restrict involvement in daily activities, social interactions, and community participation, while also emphasizing the active role they played in managing these experiences. In a sample of people with serious mental illness, Andersson et al. [22] identified sensory processing difficulties as a barrier to occupational engagement; however, participants described using sensory self-management strategies and environmental modifications to support participation in meaningful activities. Similarly, Bailliard et al. [23] found that individuals with psychotic disorders viewed themselves as active agents in navigating their sensory environments, deliberately seeking sensory experiences that enhanced wellbeing while managing sensory aversions to facilitate engagement in everyday occupations.

Both studies highlighted the complexity of sensory experiences in everyday life. While Andersson et al. [22] emphasized the role of sensory regulation strategies in maintaining occupational engagement, Bailliard et al. [23] described sensory experiences as inherently multisensory and closely intertwined with personal meanings, memories, preferences, and habits. Collectively, the qualitative evidence highlighted sensory self-management, environmental adaptation, and active engagement with sensory experiences as important features of everyday participation.

## 4. Discussion

This scoping review mapped evidence from observational, quasi-experimental, and qualitative studies and found that atypical sensory processing demonstrates a consistent relationship with participation-related outcomes among adults living with mental illness. However, the strength and mechanisms of these relationships vary across population-specific factors and distinct sensory processing patterns.

Historically, participation restrictions in mental health have been attributed largely to positive and negative symptoms, cognitive impairment, and motivation deficits [39]. While these factors remain important, sensory processing may constitute an important and potentially modifiable factor contributing to participation difficulties in adults living with mental illness [8]. Across studies using the A/ASP, atypical sensory processing was consistently associated with reduced participation and poorer participation-related outcomes in adults living with mental illness. Low registration, sensory sensitivity, and sensory avoidance were the sensory processing patterns most frequently associated with lower occupational engagement, social participation difficulties, and reduced quality of life, whereas sensation seeking showed a more variable and, in some cases, facilitative relationship with participation [16,25,26,29,35]. Clinicians should also explore how sensory experiences shape participation. This requires asking not only “What symptoms prevent participation?” but also “What sensory experiences make participation difficult?”

Assessment in mental health settings should extend beyond symptom monitoring [40]. Sensory assessments such as the A/ASP or Sensory Responsiveness Questionnaire (SRQ) should be incorporated into routine occupational therapy and mental health evaluations. Individuals with comparable psychiatric symptom profiles, symptom severity, and functional capacity may experience markedly different participation barriers because of differences in sensory processing. For example, a person with sensory sensitivity may avoid supermarkets, workplaces, or public transport because everyday sensory stimuli are experienced as overwhelming. Similarly, a person with sensory avoidance may progressively withdraw from valued occupations to minimize sensory input even when their psychiatric symptoms remain stable. While sensory sensitivity and sensory avoidance may be more visible in clinical settings, low registration was also consistently associated with poorer participation outcomes. Individuals with low registration may be less likely to notice social, environmental, and occupational cues that support participation and engagement, which may contribute to challenges that cannot be entirely explained by psychiatric symptoms alone. These vignettes highlight how sensory processing may contribute uniquely to participation outcomes and support the inclusion of sensory-informed assessment within contemporary recovery-oriented and person-centred mental health practice. This perspective may identify additional intervention opportunities that are not routinely considered within symptom-focused approaches. Beyond identifying sensory processing patterns within individuals, the qualitative findings highlighted the importance of understanding sensory experiences within the environments and occupations in which they occur.

The findings of this review are consistent with the Person-Environment-Occupation (PEO) model [41], which emphasizes that participation is shaped by the dynamic interaction between personal factors, environmental contexts, and occupational demands. Participants in qualitative research [22,23] frequently described sensory difficulties as context-dependent rather than fixed individual impairments. Conceptualizing sensory challenges as emerging from dynamic interactions between the person, environment, and occupation has important implications for intervention. Rather than locating difficulties solely within the individual, simple modifications such as reducing noise, providing quiet workspaces, and offering sensory retreat areas can increase predictability, reduce sensory burden and support engagement in meaningful daily, social and occupational activities. Similarly, occupational adaptations such as altering task demands, changing the timing of activities, balancing sensory load across the day, or introducing sensory supports within occupations can facilitate participation and engagement [16,41].

Qualitative research [22,23] indicates that people may intuitively develop personal strategies to manage sensory processing challenges. Participants described using sensory regulation strategies such as spending time in nature, seeking quiet spaces, modifying sensory input, and engaging in preferred sensory experiences to manage emotions and arousal. These findings suggest that psychoeducation regarding sensory processing could enhance self-awareness and self-management. Sensory education programs may help service users to recognize their sensory patterns, identify triggers, understand links between sensory experiences and participation, and intentionally use beneficial sensory strategies. This approach is conceptually consistent with principles underpinning Ayres Sensory Integration^®^ [42,43] and sensory modulation approaches, which seek to improve adaptive responses to sensory experiences through increased awareness and regulation.

### Limitations

The interpretation of findings from this review should be considered in light of important limitations in the current evidence base. Firstly, two qualitative studies explored the lived experience of atypical sensory processing without directly measuring sensory processing patterns [22,23], limiting the ability to determine whether findings differ across specific sensory processing profiles. Secondly, several studies reported participants using broad diagnostic categories such as psychotic disorders, serious mental illness, or mental health service users [16,22,26,29], limiting understanding of diagnosis-specific relationships between sensory processing and participation. Thirdly, despite intentionally examining this phenomenon across a broad range of mental health conditions, most included studies focused on schizophrenia-spectrum disorders, highlighting a substantial lack of studies on other mental illnesses in the literature and limiting the transferability of current findings to other mental health populations.

Finally, it is important to note that evidence from sensory modulation interventions, while promising, remains preliminary and insufficient to support robust recommendations [44]. Studies have demonstrated improvements in distress, anxiety, occupational performance, social functioning, and self-management. However, sample sizes were small, methodological quality was generally low, controlled trials remain scarce, and long-term outcomes are largely unknown [44]. Therefore, sensory modulation should currently be viewed as an adjunctive approach rather than a stand-alone treatment. Clinicians should integrate sensory approaches within broader recovery-oriented programs that also address cognitive functioning, symptom management, social participation, vocational recovery, and community integration [45].

## 5. Conclusions

This scoping review found a consistent relationship between atypical sensory processing and participation-related outcomes among adults living with mental illness. Subjective assessment using the Adult/Adolescent Sensory Profile suggested that low registration, sensory sensitivity, and sensory avoidance were associated with poorer participation-related outcomes. Findings from studies using objective measures of sensory gating and visual processing suggest that sensory dysfunction may influence daily functioning through pathways involving social cognition, motivation, emotional regulation, and negative symptoms.

Several important gaps in the evidence base were identified. Research in this field would benefit from greater consistency in terminology used to describe sensory processing constructs, particularly across neuroscience, psychiatry, psychology, occupational therapy, and rehabilitation disciplines. The literature remains heavily skewed towards schizophrenia-spectrum disorders, limiting understanding of atypical sensory processing in other mental health conditions. Finally, the limited number of intervention studies highlights the need for further research to determine whether addressing atypical sensory processing can improve participation and recovery-oriented outcomes for adults living with mental illness.

## Figures and Tables

**Figure 1:**
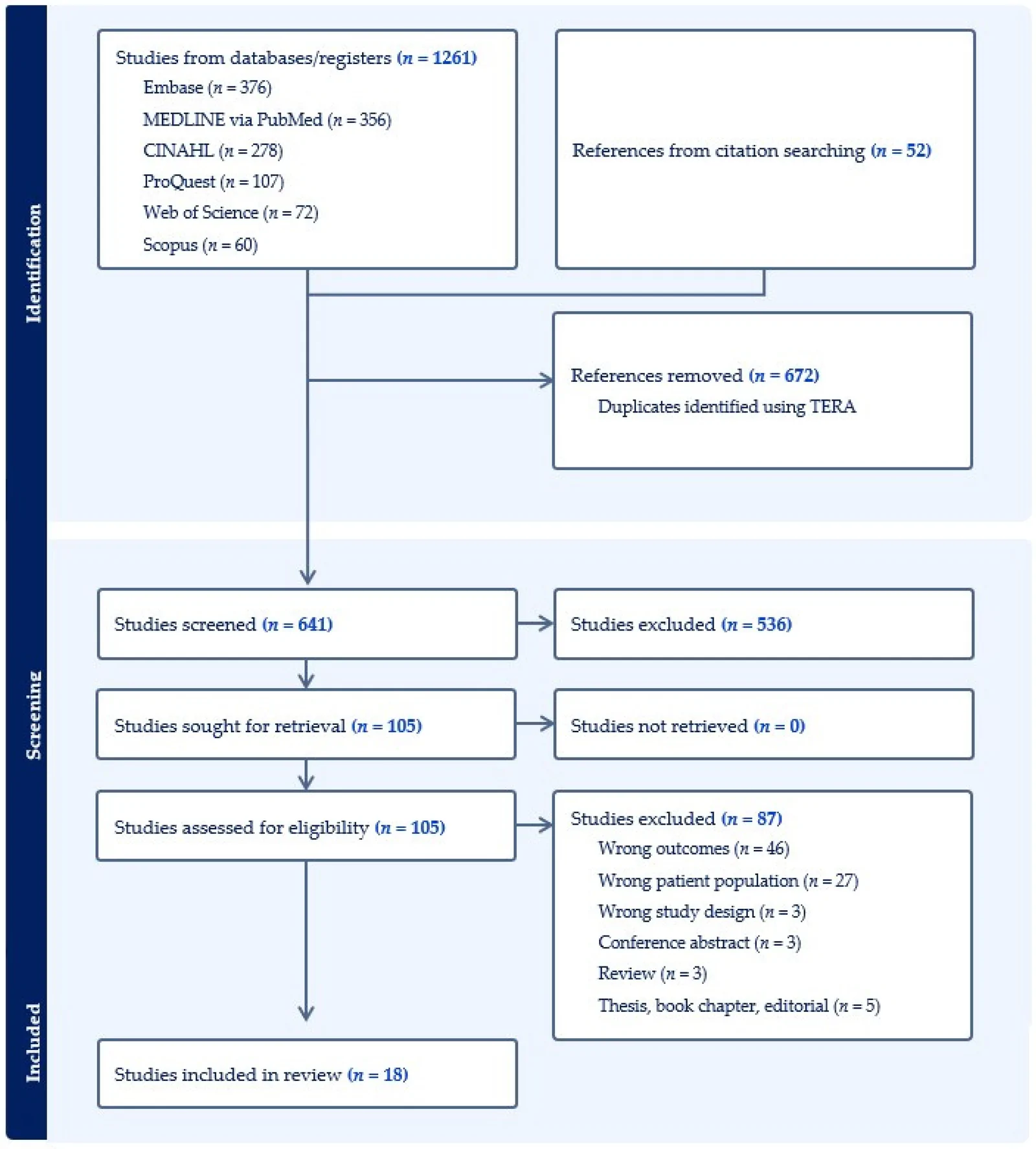
PRISMA flow diagram.

**Table 1:** Characteristics of included studies.

Author/s, Year, Country of Origin	Design	Population, Sample Size	Aim/Research Question	Measures and Outcomes
Andersson et al. (2021) [22] Sweden	Qualitative	Serious mental illness (*n* = 14)	To investigate the experience of sensory input and sensory strategies used to manage physiological and emotional arousal in daily occupations among people with serious mental illness.	**Sensory function:** Not measured, participants assumed to have atypical sensory processing. **Participation:** Qualitative exploration of occupational participation.
Bailliard et al. (2023) [23] Canada	Qualitative	Psychotic disorders (*n* = 6)	To explore how the lived sensory experiences of adults with psychotic disorders affect their daily occupations.	**Sensory function:** Not measured, participants assumed to have atypical sensory processing. **Participation:** Qualitative exploration of occupational participation.
Brittain et al. (2010) [24] England	Cross-sectional	Schizophrenia compared to healthy controls (*n* = 129)	To compare patterns of visual deficits in people with schizophrenia and healthy controls across different levels of visual processing tasks and to examine the relationship between vision and functional outcomes in people with schizophrenia.	**Sensory function:** Visual processing (low, medium, and high-level; e.g., contrast detection, global motion perception, biological motion perception) **Participation:** Role Functioning Scale
Engel-Yeger et al. (2016) [25] Italy	Cross-sectional	Major depressive disorder, Bipolar disorder-I, Bipolar disorder-II (*n* = 267)	To compare unipolar and bipolar patients with regard to their sensory processing patterns, coping strategies, and quality of life.	**Sensory function:** Adult/Adolescent Sensory Profile **Participation:** Short Form 12 Health Survey (SF-12)
Forsberg et al. (2025) [26] Sweden	Cross-sectional	Mental health service users (*n* = 97)	To investigate sensory processing patterns and their relationships to coping and occupational engagement in mental health service users.	**Sensory function:** Adult/Adolescent Sensory Profile **Participation:** Profiles of Occupational Engagement in Schizophrenia (POES)
Green et al. (2012) [27] USA	Cross-sectional	Schizophrenia or schizoaffective disorder (*n* = 191)	To evaluate theoretically based models of pathways to functional outcome starting with early visual perception.	**Sensory function:** Early visual processing (e.g., location masking, 4-dot masking) **Participation:** Role Functioning Scale
Halperin & Falk-Kessler (2020) [28] USA	Cross-sectional	Schizophrenia spectrum disorders (*n* = 17)	To examine the sensory patterns and motor and process skills of adults receiving inpatient treatment for Schizophrenia spectrum disorders.	**Sensory function:** Adult/Adolescent Sensory Profile **Participation:** Assessment of Motor and Process Skills (AMPS)
Hattori et al. (2023) [29] Japan	Cross-sectional	Serious mental illness (*n* = 30)	To investigate whether sensory processing and Respiratory Sinus Arrhythmia (RSA) are associated with impaired interpersonal relationships and social participation in people with mental illnesses.	**Sensory function:** Adult/Adolescent Sensory Profile **Participation:** Social participation scale on the WHO Disability Assessment Schedule 2.0 (WHODAS 2.0)
Light & Braff (2005) [30] USA	Cross-sectional	Schizophrenia compared to healthy controls (*n* = 50)	To determine if mismatch negativity deficits are associated with (1) clinician ratings of functioning, (2) level of independence in living situation, and (3) performance on a multidimensional functional skills assessment battery.	**Sensory function:** Mismatch negativity **Participation:** Global Assessment of Functioning; UCSD Performance-based Skills Assessment (UPSA)
Lipskaya-Velikovsky et al. (2015) [31] Israel	Cross-sectional	Schizophrenia compared to healthy controls (*n* = 81)	To explore the contributions of sensory modulation, cognition and schizophrenia symptoms to the participation of people with schizophrenia.	**Sensory function:** Sensory Responsiveness Questionnaire **Participation:** Adults Subjective Assessment of Participation (ASAP)
Machingura et al. (2022) [32] Australia	Quasi-experimental	Schizophrenia compared to wait list controls (*n* = 41)	To determine how sensory modulation interventions impact people with schizophrenia, and to establish whether people with schizophrenia have atypical sensory processing when compared with the general population in the same geographic region.	**Sensory function:** Adult/Adolescent Sensory Profile **Participation:** Canadian Occupational Performance Measure (COPM); Allen Cognitive Level Screen (ACLS); Health of the Nation Outcome Scale (HoNOS)
Micoulaud-Franchi et al. (2016) [33] France	Cross-sectional	Chronic schizophrenia (*n* = 39)	To investigate the relationship between sensory gating deficit (<50% reduction in P50 amplitude) and quality of life in patients with schizophrenia after adjusting for key confounding factors.	**Sensory function:** Auditory event-related potentials **Participation:** Schizophrenia Quality of Life Questionnaire Short Form—Clinical Practice (S-QOL 18)
Pfeiffer et al. (2014) [16] USA	Cross-sectional	Serious mental illness (*n* = 95)	To examine whether extreme responses on any of the four quadrants in the Adult/Adolescent Sensory Profile are independently related to community participation, recovery, or quality of life.	**Sensory function:** Adult/Adolescent Sensory Profile **Participation:** Temple University Community Participation Scale (TUCP)
Rassovsky et al. (2011) [34] USA	Cross-sectional	Schizophrenia (*n* = 174)	To systematically examine whether negative symptoms mediate the relationship between early visual processing and functional outcome in people with schizophrenia, or if they predict outcome independently of visual processing.	**Sensory function:** Early visual processing **Participation:** Role Functioning Scale
Sanchis-Asensi et al. (2023) [35] Spain	Cross-sectional	Schizophrenia (*n* = 99)	To explore if there is any relationship between the sensory profile and the quality of life in people with schizophrenia.	**Sensory function:** Adult/Adolescent Sensory Profile **Participation:** WHO Quality of Life—Brief (WHOQOL-BREF)
Sergi et al. (2006) [36] USA	Cross-sectional	Schizophrenia (*n* = 75)	To use structural equation modelling to examine the mediating influence of one aspect of social cognition, namely social perception, on relations between visual perception and functional status in schizophrenia.	**Sensory function:** Visual perception (e.g., visual masking) **Participation:** Role Functioning Scale
Shapira et al. (2024) [37] Israel	Cross-sectional	PTSD compared to healthy controls (*n* = 61)	To delineate the objective and subjective dimensions of participation across a range of areas in comparison to health controls, and to comprehensively investigate personal and environmental factors that impede participation among individuals with PTSD	**Sensory function:** Adult/Adolescent Sensory Profile **Participation:** UCSD Performance-based Skills Assessment (UPSA); Adults Subjective Assessment of Participation (ASAP)
Wallis et al. (2018) [38] New Zealand	Quasi-experimental	Anxiety (*n* = 4)	To examine whether a 6-week sensory modulation intervention would reduce anxiety and increase participation and improve occupational performance, and whether any changes would be sustained, post intervention, for 1 month.	**Sensory function:** Adult/Adolescent Sensory Profile **Participation:** Canadian Occupational Performance Measure (COPM); WHO Disability Assessment Schedule 2.0 (WHODAS 2.0)

## Data Availability

No new data were created or analyzed in this study. Data sharing is not applicable to this article.

## Abbreviations

The following abbreviations are used in this manuscript:

A/ASP	Adult/Adolescent Sensory Profile
PEO	Person Environment Occupation
SRQ	Sensory Responsiveness Questionnaire
QoL	Quality of Life
ICF	International Classification of Functioning Disability and Health
